# Current advances and future directions in targeting histone demethylases for cancer therapy

**DOI:** 10.1016/j.mocell.2025.100192

**Published:** 2025-02-10

**Authors:** June-Ha Shin, Hye-Been Yoo, Jae-Seok Roe

**Affiliations:** Department of Biochemistry, College of Life Science and Biotechnology, Yonsei University, Seoul, Republic of Korea

**Keywords:** Cancer, Epigenetics, Histone modification, Lysine demethylase

## Abstract

Epigenetic regulators, known as “writers,” erasers,” and “readers,” are essential for controlling gene expression by adding, removing, or recognizing post-translational modifications to histone tails, respectively. These regulators significantly affect genes involved in cancer initiation and maintenance. Recently, several clinical strategies targeting these epigenetic enzymes have emerged and some trials have demonstrated promising results for cancer treatment. Histone lysine demethylases (KDMs) yield distinct transcriptional outcomes that depend on the position of the methylated lysine and the specific genotype or lineage of the cancer cells. Due to their diverse roles in transcription, KDMs offer valuable opportunities for precision oncology, allowing treatments to be tailored to meet individual patient needs. This review emphasizes our current understanding of the functional relationship between KDMs and cancer as well as the development and application of small-molecule compounds that target KDMs.

## INTRODUCTION

Epigenetic alterations in histone tails, enable cells to dynamically adjust gene expression (Jones and Baylin, 2007). Unlike traditional genetic mutations, which cause permanent changes in the genome, epigenetic modifications lead to reversible shifts in gene expression. These epigenetic modifications affect the chemical properties of nucleosomes, further modulating their compaction and influencing transcription factor recruitment. Specifically, lysine methylation of histone tails is linked to changes in chromatin structure, which regulates the accessibility of transcription factors to regulatory regions (Bannister and Kouzarides, 2011). Moreover, this modification can drive phenotypic changes and influence cell fate (Dambacher et al., 2010). Therefore, aberrant modifications can trigger irregular cell growth and the dysregulation of cellular identity, resulting in neoplastic transformation (Chi et al., 2010, Feinberg et al., 2006, Muntean and Hess, 2009, Roy and Hebrok, 2015, Seligson et al., 2005). Therefore, a thorough understanding of lysine methylation regulators is essential.

**Fig. 1:**
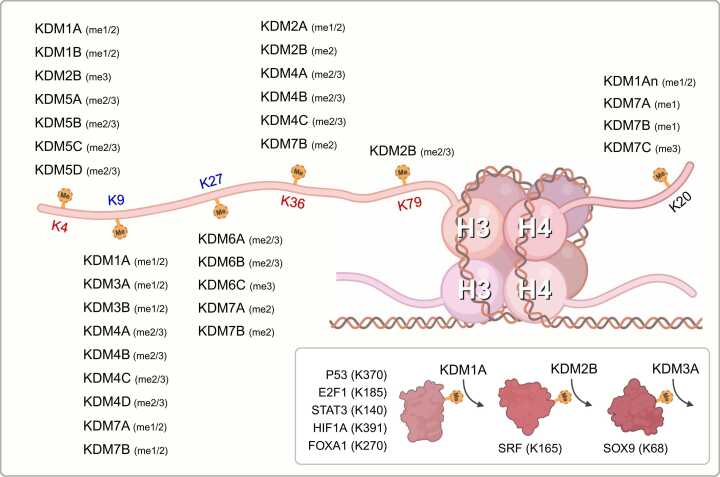
KDMs play a role in lysine demethylation of the specific substrates. Each lysine residue is categorized by distinct colors (red, transcriptionally active marks; blue, transcriptionally repressive marks; black, uncertain marks in transcription). KDMs in the lower-right box indicate their activity on methylated lysine of nonhistone substrates.

**Fig. 2:**
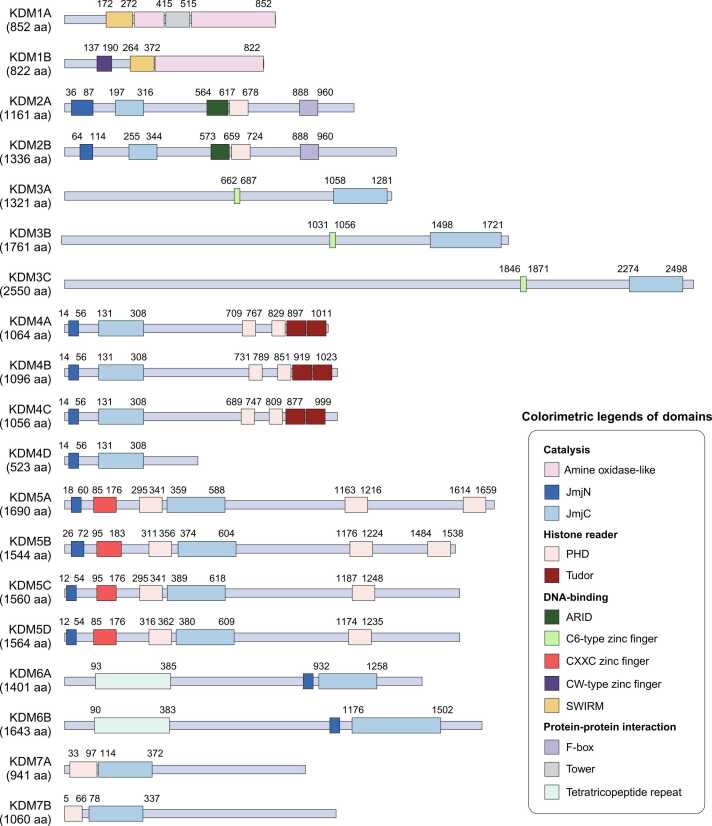
KDMs are composed of various combinations of functional domain that responsible for demethylase activity, recognizing histone modification, binding to DNA, and protein-protein interaction.

The discovery of KDM1A/LSD1, a demethylase for both mono- and dimethylation of histone H3 lysine 4 (Forneris et al., 2005, Shi et al., 2004), led to the experimental validation of various lysine demethylases (KDMs) as critical regulators of histone modification (Fig. 1). Importantly, several studies have demonstrated that dysfunctional KDMs are associated with cancer progression. Structurally, each KDM contains a binding pocket that allows enzymes to interact with other cofactors or ligands, making them potential targets for drug development (Fig. 2). Therefore, new strategies for modulating these epigenetic enzymes are being explored for targeted cancer therapies. In this review, we present the current knowledge on the biological significance of KDMs in cancer as well as on the application of KDM modulators in cancer treatment.

## GENERAL SCHEME OF KDMS

The KDM family is responsible for demethylating both active and repressive histone codes. Demethylation leads to changes in gene transcription, depending on which lysine methylation is removed. Besides, some KDMs, such as KDM1A, KDM2B, KDM4, and KDM7, are involved in the demethylation of multiple lysine residues due to their structural properties or variations in co-effector proteins (Ismail et al., 2018, Ramanan et al., 2020). Recent studies have shown that some KDMs regulate the post-translational modifications of nonhistone proteins. However, the functions of KDMs that target nonhistone substrates remain largely unknown. Therefore, we primarily focused on how KDMs mediate gene expression by demethylating lysine residues in histone tails.

### Histone Methylation in Transcriptionally Active Genes

#### H3K4me (KDM1/KDM2B/KDM5)

KDM1 family members are H3K4me1/2 demethylases whose action leads to transcriptional repression; KDM1 members use their amine oxidase-like domain (AOD) and FAD^+^—as cofactor—for demethylation. To properly recognize nucleosomes, KDM1A forms complexes with other chromatin remodelers and DNA-binding proteins, such as CoREST and NuRD (Lee et al., 2005, Shi et al., 2004). Similarly, KDM1B interacts with NPAC/GLYR1 to efficiently bind to nucleosomes (Chen et al., 2013, Ciccone et al., 2009, Fang et al., 2010, Fang et al., 2013). KDM2B and KDM5 family members play enzymatic roles on H3K4me3 and H3K4me2/3, respectively; their activity depends on α-ketoglutarate as the cofactor of the Jumonji domain (JmjC) (Christensen et al., 2007, Frescas et al., 2007, Iwase et al., 2007, Tzatsos et al., 2009). Additionally, KDM2B and KDM5 family members have PHD domains that recognize the H3K4 histone tail and facilitate demethylation of H3K4me2/3 (Klein et al., 2014, Lee et al., 2007a, Torres et al., 2015).

#### H3K36me (KDM2/KDM4/KDM7B)

Methylation of histone H3 lysine 36 is erased by several JmjC-containing KDMs. KDM2A demethylates H3K36me1/2, whereas KDM2B only targets H3K36me2 (Tsukada et al., 2006, Tzatsos et al., 2009). The KDM4 family bivalently influences gene expression by eliminating (erasing) both active and repressive markers (H3K36me2/3 and H3K9me2/3). The role of the KDM4 family in regulating H3K36me2/3 has been demonstrated by in vitro assays using *Saccharomyces cerevisiae*. Subsequent studies have demonstrated KDM4 member’s transcriptional repression functions (Labbe et al., 2013, Ng et al., 2007, Tu et al., 2007). In addition, using mass spectrometry screening, KDM7B was reported to selectively demethylate H3K36me2 (Loenarz et al., 2010).

#### H3K79me (KDM2B)

KDM2B has been reported as demethylase of di- and trimethylated lysine 79 of histone H3. A recent study, using in vitro assays such as formaldehyde dehydrogenase assay and radioactive assay, showed its demethylase activity of H3K79me2/3, but not H3K79me1 (Kang et al., 2018).

### Histone Methylation in Transcriptionally Inactive Genes

#### H3K9me (KDM1A/KDM3/KDM4/KDM7)

KDM1A demethylates H3K9me1/2 to transactivate the target genes of the hormone receptors (Carnesecchi et al., 2017, Metzger et al., 2005), and a unique isoform of KDM1A (LSD1+8a) interacts with SVIL to demethylate H3K9me2 (Laurent et al., 2015). Using in vitro demethylation assays, a recent study confirmed that KDM3A and KDM3B demethylate H3K9me1/2 (Gray et al., 2023), while the demethylase function on H3K9me2/3 KDM4 family members has also been demonstrated (Cloos et al., 2006, Ng et al., 2007). However, under the androgen receptor (AR)-dependent expression, KDM4C only targets H3K9me3, and does not affect H3K9me1/2 (Whetstine et al., 2006, Wissmann et al., 2007). Regarding the KDM7 family, both KDM7A and KDM7B have been shown to demethylate H3K9me1/2 (Liu et al., 2010, Loenarz et al., 2010, Qi et al., 2010). Furthermore, in the presence of H3K4me3, these enzymes more efficiently demethylate H3K9me1/2 due to their PHD domains, which bind favorably to H3K4me3 (Horton et al., 2010, Rissi et al., 2019).

#### H3K27me (KDM6/KDM7)

Both the KDM6/7 families are involved in the demethylation of H3K27me (Agger et al., 2007, De Santa et al., 2007). KDM6A and KDM6B target H3K27me2/3, whereas KDM6C acts only on H3K27me3 demethylation (Walport et al., 2014). In addition, KDM7A and KDM7B are H3K27me2 demethylases (Liu et al., 2010, Qi et al., 2010). Notably, KDM6A and KDM7A show more efficient demethylase activity on H3K27me when H3K4me is enriched (Horton et al., 2010, Lee et al., 2007b).

### Other Substrates

#### H4K20me (KDM1An, KDM7)

In contrast to the role of other histone lysine methylation marks, the influence of H4K20me on gene expressions is still controversial (Schotta et al., 2004, Yang et al., 2016). KDM1An, a unique spliced variant of KDM1A, is specifically expressed in neuronal cells, and targets H4K20me1/2 (Wang et al., 2015), whereas KDM7A and KDM7B demethylate H4K20me1 and KDM7C demethylates H4K20me3 (Liu et al., 2010, Stender et al., 2012).

#### Nonhistone Substrates

KDMs have a high specificity for lysine residues in histone tails, but few KDMs recognize and demethylate nonhistone substrates (Fig. 1). KDM1A, which mainly shows demethylase activity on H3K4me1/2 or H3K9me1/2, recognizes various transcription factors, including P53-K370, E2F1-K185, STAT3-K140, HIF1A-K391, and FOXA1-K270, and either affects their stability or function (Gao et al., 2020, Huang et al., 2007a, Kontaki and Talianidis, 2010, Lee et al., 2017, Yang et al., 2010). Furthermore, KDM2B demethylates the lysine 165 residue of SRF, SET7-mediated methylation, thereby regulating the expression of SRF-dependent genes (Kwon et al., 2021). KDM3A, another enzyme that targets nonhistone substrate, is involved in the demethylation of the lysine 68 residue of SOX9, attenuating the ubiquitination of this protein (Sun et al., 2023).

## IMPORTANCE OF KDMS IN HUMAN CANCER

**Fig. 3:**
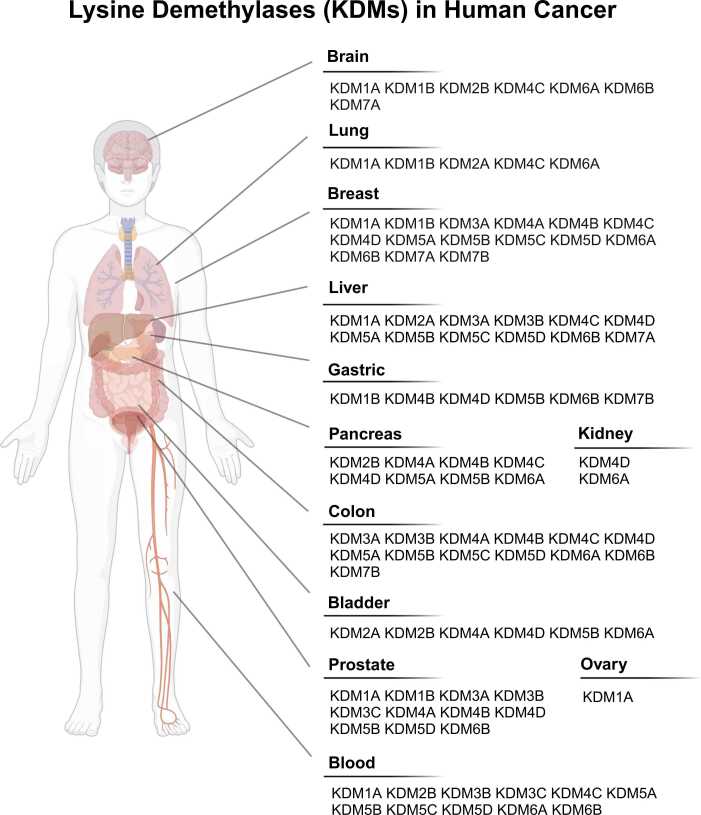
KDMs are involved in progression of various human cancers.

To date, various studies have demonstrated the relationship between KDMs and cancer progression. These enzymes play crucial roles in promoting or suppressing important cancer-related genes. In this section, we summarize the current understanding of the role of KDMs in tumorigenesis (Fig. 3).

### The KDM1 Family

KDM1A is supposed to be a key oncogene in various cancers. It regulates epithelial-mesenchymal phenotypic changes by repressing the expression of epithelial markers (Lin et al., 2010, Luo et al., 2016). KDM1A also regulates cell proliferation-related genes and pathways, such as *CDKN1A*, *ERBB2*, *CCNA2*, and the TGF-β1 signaling pathway, in breast cancer (Kim et al., 2017, Lim et al., 2010, Wang et al., 2009). In prostate cancer, KDM1A regulates the transcription of AR target genes by demethylating H3K9me (Kahl et al., 2006, Metzger et al., 2005). Moreover, KDM1A acts as a coactivator with FOXA1 to upregulate AR-related gene transcription (Cai et al., 2014). Furthermore, KDM1A has been implicated in multiple cancers, such as leukemia, liver cancer, ovarian cancer, and neuroblastoma (Harris et al., 2012, Lei et al., 2015, Schulte et al., 2009, Shao et al., 2015). KDM1B, another AOD-containing demethylase, is involved in the proliferation of small-cell lung cancer by regulating the expression of *TFPI-2* (Cao et al., 2018). In breast cancer cells, the overexpression of KDM1B leads to the upregulation of the pluripotent stem cell markers *NANOG* and *SOX2* (Chen et al., 2017). Moreover, KDM1B enables glioma cells to proliferate in a hypoxic environment via the activation of the HIF1 pathway (Hu et al., 2016). Several cohort databases have also shown a positive correlation between the expression level of KDM1B and the progression of prostate and gastric cancers (Huang and Yang, 2019, Tang et al., 2022).

### The KDM2 Family

KDM2A suppresses histone deacetylase 3 in non–small-cell lung cancer cells, enhancing cancer cell proliferation and invasiveness by demethylating of H3K36me2 at histone deacetylase 3 promoter region (Dhar et al., 2014). KDM2A also promotes the proliferation and migration of esophageal squamous cell carcinoma cells (Wang et al., 2022). Moreover, in bladder cancer, KDM2A suppresses the expression of *RARRES3*, leading to cell proliferation, invasion, and spheroid formation (Lu et al., 2022). KDM2B regulates FGF-2-mediated cell proliferation, migration, and angiogenesis by coupling with the histone H3K27 methyltransferase EZH2 (Kottakis et al., 2011). Moreover, KDM2B plays oncogenic roles by silencing *CDKN2B* expression during the progression of leukemia development and maintenance (He et al., 2011). KDM2B is also involved in the regulation of apoptosis in glioblastoma cells (Kurt et al., 2017) and the expression of epithelial-mesenchymal transition-related genes, such as *CDH1*, *miR-200a*, and *CGN*, in pancreatic cancer (Wanna-Udom et al., 2021).

### The KDM3 Family

KDM3A enhances growth ability by controlling the expression of *c-myc* in prostate cancer (Fan et al., 2016). Likewise, the expression level of KDM3A is associated with poor survival rates in patients with hepatocarcinoma (Lin et al., 2023), and the ablation of KDM3A and KDM3B reduces the proliferation of hepatocarcinoma cells (An et al., 2019, Yamada et al., 2012). KDM3A and KDM3B also allow cancer cells to survive under hypoxic conditions via the upregulation of *HIF1A* (Beyer et al., 2008, Guo et al., 2011). Furthermore, several studies have reported that the KDM3 family is associated with various cancers, such as AR-negative prostate cancer (Yoshihama et al., 2021), breast cancer (Wade et al., 2015), colorectal cancer (Li et al., 2017, Uemura et al., 2010), and leukemia (Kim et al., 2012).

### The KDM4 Family

In prostate cancer, members of the KDM4 family demethylate H3K9me in the promoter regions of AR-related genes (Coffey et al., 2013, Shin and Janknecht, 2007). Additionally, KDM4B and KDM4C promote tumor growth in multiple cancers, including brain, colon, and gastric cancers (Fu et al., 2018, Lee et al., 2021, Li et al., 2011, Shi et al., 2011). KDM4 family members not only act on H3K9me but also on H3K36me. KDM4A suppresses cell growth by inducing DNA damage via RFXAP-dependent H3K36 demethylation in pancreatic cancer cells (Ding et al., 2020). In liver cancer, KDM4C regulates cell migration by erasing H3K36me of *CXCL2* promoter region (Zeng et al., 2023). On the other hand, KDM4C collaborates with HIF1A to reprogram metabolic processes and promote lung metastasis (Luo et al., 2012). Consistently, KDM4D also regulates the HIF pathway in several gastrointestinal tumors (Hu et al., 2018, Peng et al., 2020). KDM4D interacts with β-catenin, leading to decreased H3K9me3 levels at the promoters of *EPCAM* and *SOX9*, which subsequently increases their expressions (Deng et al., 2021). The role of the KDM4D-β-catenin interaction has also been investigated in colorectal cancer (Peng et al., 2019).

### The KDM5 Family

The function of KDM5 family members is well-defined in breast cancer. KDM5A is involved in cell cycle-associated gene expression via PI3K-/AKT-mediated phosphorylation, regulating nuclear localization of KDM5A (Spangle et al., 2016). Similarly, KDM5B suppresses the expression of proliferation-related genes, including *CAV1*, *HOXA5,* and *BRCA1*, by modulating H3K4me3 levels (Yamane et al., 2007). The KDM5 family members also play a role in the immune system of breast cancer. For example, KDM5B regulates the expression of inflammation- and luminal-related genes, such as *ESRP1*, *ENAH*, and *CD44* (Klein et al., 2014, Yamamoto et al., 2014). In addition, KDM5C suppresses type I interferons and their target genes to promote tumorigenesis in ERα-positive breast cancer (Shen et al., 2021). As suggested for breast cancer, KDM5 family members have emerged as therapeutic targets for various cancers (Hayami et al., 2010, Li et al., 2023, Wang et al., 2016a, Wang et al., 2023, Wang et al., 2016b, Wang et al., 2024). KDM5A targets H3K4me2 in the promoter region of *MPC-1*, affecting cell growth, invasion, migration, and stemness in pancreatic cancer (Cui et al., 2020). Furthermore, KDM5B induces prostate cancer progression by regulating the transcription of AR-related genes (Xiang et al., 2007).

### The KDM6 Family

KDM6A functions in a sex-dependent manner because the gene is located on the X chromosome. Therefore, somatic mutations in KDM6A are commonly found in noninvasive female tumors (Hurst et al., 2017, Kaneko and Li, 2018). In bladder cancer, KDM6A inhibits colony formation by downregulating *FGFR3* expression (Barrows et al., 2020). Furthermore, loss-of-function mutations in KDM6A render bladder cancer cells susceptible to EZH2 inhibitors, which drive cell differentiation and death (Ramakrishnan et al., 2019). KDM6A is also crucial for determining cancer subtypes. In pancreatic cancer, KDM6A inhibition induces the transition of progenitor-like subtype cells into a squamous-like subtype (Andricovich et al., 2018). In addition, together with HNF1A, KDM6A activates the epithelial transcriptional program (Kalisz et al., 2020). Besides, KDM6A is involved in the plasticity of small-cell lung cancer SCLC-A and SCLC-N subtypes (Duplaquet et al., 2023), and regulates cancer progression and metastasis via the TGF-β and Wnt pathways (Leng et al., 2020, Terashima et al., 2017). KDM6A also influences cancer cell proliferation and migration in breast cancer, kidney cancer, and multiple myeloma (Choi et al., 2015, Ezponda et al., 2017, Song et al., 2023). KDM6B promotes the oncogenic CDK4/6-pRB-E2F pathway in neuroblastoma by enhancing the chromatin accessibility of E2F target genes and *MYCN* (D'Oto et al., 2021). KDM6B also upregulates cyclin D1 transcription in prostate cancer (Cao et al., 2021). Moreover, KDM6B induces the expression of the mesenchymal genes *SNAI1* and *CXCR4*, which contribute to the metastasis of breast and gastric cancers (Liu et al., 2022, Ramadoss et al., 2012), and its expression is associated with poor outcomes in patients with gastric cancer (Xu et al., 2019).

### The KDM7 Family

Under nutrient-depleted conditions, KDM7A downregulates angiogenesis-regulating genes such as *VEGF-α* (Osawa et al., 2011). In contrast to its antitumorigenic role, KDM7A inhibits apoptosis by upregulating *BCL2* levels and promotes cell migration and invasion of breast cancer (Meng et al., 2020, Zhang et al., 2021). Additionally, in HER2-negative gastric cancer, KDM7B interacts with c-Jun and facilitates cancer progression by activating the PKCα-Src-PTEN pathway (Tseng et al., 2020).

## KDMS AS THERAPEUTIC TARGETS

Molecular inhibitors of KDMs weaken their enzymatic functions by disturbing the interactions with their corresponding cofactors, such as FAD^+^ and 2-oxoglutarate, or ligands. Recent studies have reported that these inhibitors exert antitumorigenic effects across multiple cancer lineages (Fig. 3). In this section, we discuss current progress in the application of KDM inhibitors and their therapeutic potential against a variety of cancers (Table 1).

**Table 1:** Experimental and clinical information on KDM-targeting drugs for treating human cancer

Drug	Target	Applications in cancer (PMID)	Clinical phase (indication)
*Targeting AOD-containing KDMs*			
Tranylcypromine (TCP)	KDM1A	Reactivates the differentiation pathway via retinoic acid receptor alpha (RARα)-mediated transcription in AML (PMID: 22406747)	Phase 1/2 (AML)
Iadademstat (ORY-1001)	KDM1A	Increases the expression of differentiation-related genes in AML (PMID: 29502954)	Phase 1 (AML) Phase 1/2 (SCLC)
Bomedemstat (IMG-7289)	KDM1A	Upregulates *MHC-I* expression, resulting in sensitivity to PD-1 inhibition in SCLC (PMID: 35920742)	Phase 1/2 (SCLC, AML) Phase 2/3 (thrombocythemia)
GSK-2879552	KDM1A	Depletes growth activity in SCLC, enriching hypomethylated DNA (PMID: 26175415)	Phase 1 (SCLC, AML)
GSK-LSD1	KDM1A	Inhibits GFI-mediated oncogenic transformation and reactivates neuronal differentiation in GFI-driven medulloblastoma (PMID: 30659187)	Preclinical
INCB059872	KDM1A	Impairs platelet production via accumulation of early megakaryocyte population in AML (PMID: 32422235)	Phase 1 (ES)
CC-90011	KDM1A	Induces hematopoietic differentiation and growth arrest in AML (PMID: 33034194)	Phase 1 (SCLC, lymphoma)
Seclidemstat (SP-2577)	KDM1A	Promotes antitumor immunity in SWI-/SNF-mutated complexes in ovarian cancer (PMID: 32649682)	Phase 1/2 (ES)
SP-2509	KDM1A	Elicits tumor immunity via immune checkpoint blockade in breast cancer (PMID: 30111819)	Preclinical
CBB1007	KDM1A	Reduces *Sox2* expression and promotes G1 cell cycle arrest in various carcinomas (PMID: 24139802)	Preclinical
*Targeting JmjC-containing KDMs*			
CBA-1	KDM3A/3B	Suppresses the Wnt signaling pathway in CRC (PMID: 33305174)	Preclinical
JDI-4/12/16	KDM3B/3C	Induces apoptosis and hematopoietic differentiation in AML (PMID: 31271662)	Preclinical
SD70	KDM4C	Inhibits the expression of MLL target genes and shows antitumorigenic effects in AML (PMID: 26766589)	Preclinical
Caffeic acid	KDM4C	Impairs NOTCH1 signaling pathways, resulting in loss of stemness in ESCC (PMID: 31031809)	Phase 3 (ESCC)
KDM4D-IN-1	KDM4D	Suppresses tumor angiogenesis via JAG1 signaling in RCC (PMID: 34667158)	Preclinical
QC6352	Pan-KDM4	Displays antiproliferative effects in breast and colon PDX models (PMID: 28835804)	Preclinical
TACH-101	Pan-KDM4	Induces apoptosis and cell cycle arrest and reduces tumor-initiating cell populations in multiple gastrointestinal cancers (PMID: 37067993)	Phase 1 (solid)
CPI-455	Pan-KDM5	Enhances chemosensitivity to cisplatin and apoptosis by modulating the ROCK1/PTEN/AKT pathway in liver cancer (PMID: 36566915)	Preclinical
KDOAM-20 (KDM5-C49)	Pan-KDM5	Induces interferon response via *STING* expression and leads to increased accumulation of cytosolic DNA in breast cancer (PMID: 30080846)	Preclinical
KDOAM-25 (KDM5-C70)	Pan-KDM5	Induces G1 cell cycle arrest in multiple myeloma cells (PMID: 28262558)	Preclinical
Compound 1	KDM5A	Induces cell cycle arrest and senescence in breast cancer (PMID: 30650517)	Preclinical
Compound 27ab	KDM5B	Inhibits proliferation and migration in gastric carcinoma (PMID: 32155529)	Preclinical
GSK-J1	Pan-KDM6	Blocks cell proliferation and induces apoptosis and senescence in HNSCC (PMID: 31848446)	Preclinical
GSK-J4	KDM6A/6B	Reduces proliferative activity in brainstem glioma (PMID: 25401693) and AML (PMID: 29594337)	Preclinical
KDM2A/7A-IN-1	KDM2A/7A	N/A	Preclinical
IOX1 (5-c-8HQ)	KDM2A/3A/4C/4D/6B	Reduces self-renewal and tumorigenic activities in liver cancer stem-like cells (PMID: 33434575)	Preclinical
JIB-04	Pan-KDM4/5/6	Inhibits the Wnt/β-catenin signaling pathway in CRC stem cells (PMID: 29700375)	Preclinical

AOD, amine oxidase-like domain; AML, acute myeloid leukemia; CBA-1, carboxamide-substituted benzhydryl amine; CRC, colorectal cancer; ES, Ewing sarcoma; ESCC, esophageal squamous cell carcinoma; HNSCC, head and neck squamous cell cancer; N/A, not applicable; PDX, patient-derived xenograft; RCC, renal cell carcinoma; SCLC, small-cell lung cancer.

### The KDM1 Family

Several inhibitors of the KDM1 family are currently undergoing clinical trials for cancer treatment. Since the catalytic activity of the KDM1 family is dependent on AOD, the rationale for developing new inhibitors is mainly focused on targeting FAD^+^ reduction. Tranylcypromine (TCP), which was first developed to treat depressive disorders and later identified as a KDM1 inhibitor, covalently binds to FAD^+^, resulting in the reactivation of the expression of silenced genes (Huang et al., 2007b, Mimasu et al., 2008). Accordingly, various TCP derivatives have been developed for use in cancer research. For example, in small-cell lung cancer cells, GSK-2879552 causes growth depletion in hypomethylated DNA-enriched cells, while IMG-7289 sensitizes cells to immune checkpoint blockade and T-cell-driven cell death (Hiatt et al., 2022, Mohammad et al., 2015). Besides, ORY-1001 reduced growth in a xenograft model of acute myeloid leukemia. Furthermore, a patient-derived xenograft (PDX) model showed that ORY-1001-treated mice survived longer than untreated mice (Maes et al., 2018).

Unlike TCP variants that form strong covalent bonds with FAD^+^, other chemical compounds induce the reversible inhibition of KDM1A. CC-90011, an orally active inhibitor of KDM1A, displays on-target induction of differentiation markers in in vitro cell line models and shows antitumor efficacy in PDX models of small-cell lung cancer (Kanouni et al., 2020). Moreover, a recent clinical trial indicates that patients with advanced solid tumors or relapsed/refractory marginal zone lymphoma have positive effects upon treatment with CC-90011 (Hollebecque et al., 2022). SP-2577, which is currently undergoing a clinical trial for Ewing sarcoma, activates T-cell infiltration by upregulating the expression of immune-related chemokines (especially *PD-L1*) in ovarian cancer cells harboring a mutated SWI/SNF complex (Soldi et al., 2020).

### The KDM3 Family

Carboxamide-substituted benzhydryl amine (CBA-1), a biotinylated variant of CBAs, was identified as a selective inhibitor of KDM3A. In colorectal cancer, treatment with CBA-1 increases global H3K9me2 levels, reducing the expression of Wnt target genes and the proliferation of organoids (Zhang et al., 2020b). Another study demonstrated that JDI-4/12/16 are inhibitors of KDM3C by virtually screening 183,284 small-molecule modulators. Compared with JDI-4/12, JDI-16 exhibited more potent efficacy in promoting hematopoietic cell differentiation. Furthermore, JDI-16 showed synergistic effects in repressing the proliferation of acute myeloid leukemia cells when such JDI-16 was treated with all-trans retinoic acid (Xu et al., 2020).

### The KDM4 Family

8-hydroxyquinoline (8HQ) is an inhibitor of 2-oxoglutarate oxygenase, which targets multiple KDMs. As a derivative of 8HQ, SD70 is a selective KDM4C inhibitor. In MLL-fusion acute myeloid leukemia, SD70 reduced the proliferative ability and showed synergistic effects when simultaneously used with AMI-408 (a PRMT1 inhibitor) or MI-503 (a menin inhibitor) for treatment (Cheung et al., 2016, Zhu et al., 2024). Caffeic acid, another inhibitor of KDM4C, is a natural compound that exerts antitumorigenic effects by suppressing NOTCH1 signaling pathways in esophageal squamous cell carcinoma and glioma (Jia et al., 2019, Xiao et al., 2021). KDM4D-IN-1 broadly inhibits JmjC-containing KDMs but is more selective to KDM4D than to KDM2B, KDM3B, and KDM5A (Fang et al., 2017). Treatment with KDM4D-IN-1 reduces the invasiveness of several renal cell carcinoma lines by suppressing the JAG1 pathway (Yan et al., 2021). Additionally, TACH-101, a first-in-class pan-KDM4 inhibitor that is undergoing clinical trials for solid cancers (phase I), induces growth arrest in multiple cell lines and organoids derived from various histologies (Chandhasin et al., 2023).

### The KDM5 Family

Pyrazol-pyridine compounds are among the basic structures of the inhibitors of JmjC-containing KDMs (KDM2-7). CPI-455, a derivative of these compounds, has topological advantages for inhibiting KDM5 family members, leading to a reduction in the viability of multiple drug-tolerant cancer cells (Vinogradova et al., 2016). In liver cancer, CPI-455 also induces apoptotic cell death and enhances vulnerability to cisplatin (Fang et al., 2023). KDOAM-X (KDM5-CX), an analog of 2,4-pyridine dicarboxylic acid, is a pan-KDM5 inhibitor. Besides, in various breast cancer cell lines, KDOAM-20 (KDM5-C49) has shown potent efficacy in tumor suppression with global upregulation of H3K4me3 (Hinohara et al., 2018). KDOAM-25 (KDM5-C70) induces G1-phase cell cycle arrest in multiple myeloma cell lines (Horton et al., 2016, Johansson et al., 2016, Tumber et al., 2017). In addition, cyclopenta[*c*] chromen derivative (compound 1) and pyrazole derivative (compound 27ab) are considered candidate inhibitors of KDM5A and KDM5B, respectively (Yang et al., 2019, Zhao et al., 2020).

### Other KDM Families

GSK-J1 and GSK-J4, which derived from a large-scale compound screening, can bind the catalytic site of KDM6 family members, resulting in the interruption of cofactor binding. GSK-J1 regulates TNF-alpha production in human primary macrophages by demethylating H3K27me3 of *TNFA* transcription start sites (Kruidenier et al., 2012). Moreover, combinatorial treatments with GSK-J1 and TCP synergistically inhibit tumor progression in head and neck squamous cell carcinoma (Zhang et al., 2020a). GSK-J4 increases global H3K27me3 levels, resulting in antitumorigenic effects on multiple brainstem gliomas in both in vitro and in vivo experiments (Hashizume et al., 2014). GSK-J4 also exerts synergistic effects with cytosine arabinoside by regulating *HOX* genes in acute myeloid leukemia (Li et al., 2018).

Although cancer-associated studies have not yet been conducted, KDM2A/7A-IN-1 is considered an inhibitor of KDM2A and KDM7A (Gerken et al., 2017). Besides, as a multiple KDM-targeting inhibitor, IOX1 (5-c-8HQ) shows broad-spectrum inhibition of JmjC demethylases, including KDM2A/3A/4C/4D/6B (Schiller et al., 2014). In osteosarcoma, IOX1 suppresses metastasis and sensitizes cisplatin-resistant cells to cisplatin (Chang et al., 2023). In addition, JIB-04 is a pan-KDM4/5/6 inhibitor that exhibits tumor-suppressive activity without general toxicity. Treatment with JIB-04 induces suppression of the Wnt/β-catenin signaling pathways, repressing the self-renewal and stemness of colorectal cancer stem cells (Kim et al., 2018).

## DISCUSSION

Regulation of gene expression by specific epigenetic enzyme modulation is advantageous because it does not cause any permanent changes to the genome. Recent studies have shown that epigenetic regulator inhibitors can be used in personalized therapies. For example, protein arginine methyltransferase 5 (PRMT5) inhibitors can be used in patients with deletions in the methylthioadenosine phosphorylase gene (Kryukov et al., 2016).

To this date, histone demethylases (KDMs) have emerged as promising candidates for epigenetic cancer therapy with advanced techniques. PROTAC (proteolysis-targeting chimera) molecules have recently been employed to selectively degrade target KDMs (Guan et al., 2024). For example, IOX1-PROTAC, CRBN-recruiting PROTAC molecule synthesized using IOX1, effectively degraded KDM3 family and suppressed the self-renewal ability of colorectal cancer stem cells by disrupting Wnt/β-catenin signaling (Zaman et al., 2024). As KDMs are also implicated in various diseases, including neurological, immune, and metabolic disorders, these advancements provide opportunities to broaden the scope of therapeutic applications (Mokou et al., 2019, Park et al., 2014, Zhou et al., 2020). However, several challenges remain, such as the unclear mechanisms of action of some inhibitors and the development of methods to minimize unintended activities in nontarget tissues. Further research is needed to fully understand how the therapeutic potential of KDM inhibitors can be enhanced.

In conclusion, this review provides guidance on the appropriate directions for advancing therapies targeting KDMs.

## FUNDING AND SUPPORT

This work was supported by a National Research Foundation of Korea grant funded by the Korean Government (RS-2024-00338254). All authors were also supported by the Brain Korea 21 FOUR Program.

## AUTHOR CONTRIBUTIONS

**Shin June-Ha:** Writing – review & editing, writing – original draft, conceptualization. **Roe Jae-Seok:** Writing – review & editing, writing – original draft, supervision, funding acquisition. **Yoo Hye-Been:** Writing – review & editing, writing – original draft.

## DECLARATION OF COMPETING INTERESTS

The author(s) declare that they have no competing interests.

## References

[bib1] Agger K., Cloos P.A., Christensen J., Pasini D., Rose S., Rappsilber J., Issaeva I., Canaani E., Salcini A.E., Helin K. (2007). UTX and JMJD3 are histone H3K27 demethylases involved in HOX gene regulation and development. Nature.

[bib2] An M.J., Kim D.H., Kim C.H., Kim M., Rhee S., Seo S.B., Kim J.W. (2019). Histone demethylase KDM3B regulates the transcriptional network of cell-cycle genes in hepatocarcinoma HepG2 cells. Biochem. Biophys. Res. Commun..

[bib3] Andricovich J., Perkail S., Kai Y., Casasanta N., Peng W., Tzatsos A. (2018). Loss of KDM6A activates super-enhancers to induce gender-specific squamous-like pancreatic cancer and confers sensitivity to BET inhibitors. Cancer Cell.

[bib4] Bannister A.J., Kouzarides T. (2011). Regulation of chromatin by histone modifications. Cell Res..

[bib5] Barrows D., Feng L., Carroll T.S., Allis C.D. (2020). Loss of UTX/KDM6A and the activation of FGFR3 converge to regulate differentiation gene-expression programs in bladder cancer. Proc. Natl. Acad. Sci. U.S.A..

[bib6] Beyer S., Kristensen M.M., Jensen K.S., Johansen J.V., Staller P. (2008). The histone demethylases JMJD1A and JMJD2B are transcriptional targets of hypoxia-inducible factor HIF. J. Biol. Chem..

[bib7] Cai C., He H.H., Gao S., Chen S., Yu Z., Gao Y., Chen S., Chen M.W., Zhang J., Ahmed M. (2014). Lysine-specific demethylase 1 has dual functions as a major regulator of androgen receptor transcriptional activity. Cell Rep..

[bib8] Cao Y., Guo C., Yin Y., Li X., Zhou L. (2018). Lysine‑specific demethylase 2 contributes to the proliferation of small cell lung cancer by regulating the expression of TFPI‑2. Mol. Med. Rep..

[bib9] Cao Z., Shi X., Tian F., Fang Y., Wu J.B., Mrdenovic S., Nian X., Ji J., Xu H., Kong C. (2021). KDM6B is an androgen regulated gene and plays oncogenic roles by demethylating H3K27me3 at cyclin D1 promoter in prostate cancer. Cell Death Dis..

[bib10] Carnesecchi J., Forcet C., Zhang L., Tribollet V., Barenton B., Boudra R., Cerutti C., Billas I.M., Serandour A.A., Carroll J.S. (2017). ERRalpha induces H3K9 demethylation by LSD1 to promote cell invasion. Proc. Natl. Acad. Sci. U.S.A..

[bib11] Chandhasin C., Dang V., Perabo F., Del Rosario J., Chen Y.K., Filvaroff E., Stafford J.A., Clarke M. (2023). TACH101, a first-in-class pan-inhibitor of KDM4 histone demethylase. Anticancer Drugs.

[bib12] Chang S.L., Lee C.W., Yang C.Y., Lin Z.C., Peng K.T., Liu S.C., Wang S.W., Tsai H.C., Fong Y.C., Lai C.Y. (2023). IOX-1 suppresses metastasis of osteosarcoma by upregulating histone H3 lysine trimethylation. Biochem. Pharmacol..

[bib13] Chen F., Yang H., Dong Z., Fang J., Wang P., Zhu T., Gong W., Fang R., Shi Y.G., Li Z. (2013). Structural insight into substrate recognition by histone demethylase LSD2/KDM1b. Cell Res..

[bib14] Chen L., Vasilatos S.N., Qin Y., Katz T.A., Cao C., Wu H., Tasdemir N., Levine K.M., Oesterreich S., Davidson N.E. (2017). Functional characterization of lysine-specific demethylase 2 (LSD2/KDM1B) in breast cancer progression. Oncotarget.

[bib15] Cheung N., Fung T.K., Zeisig B.B., Holmes K., Rane J.K., Mowen K.A., Finn M.G., Lenhard B., Chan L.C., So C.W. (2016). Targeting aberrant epigenetic networks mediated by PRMT1 and KDM4C in acute myeloid leukemia. Cancer Cell.

[bib16] Chi P., Allis C.D., Wang G.G. (2010). Covalent histone modifications--miswritten, misinterpreted and mis-erased in human cancers. Nat. Rev. Cancer.

[bib17] Choi H.J., Park J.H., Park M., Won H.Y., Joo H.S., Lee C.H., Lee J.Y., Kong G. (2015). UTX inhibits EMT-induced breast CSC properties by epigenetic repression of EMT genes in cooperation with LSD1 and HDAC1. EMBO Rep..

[bib18] Christensen J., Agger K., Cloos P.A., Pasini D., Rose S., Sennels L., Rappsilber J., Hansen K.H., Salcini A.E., Helin K. (2007). RBP2 belongs to a family of demethylases, specific for tri-and dimethylated lysine 4 on histone 3. Cell.

[bib19] Ciccone D.N., Su H., Hevi S., Gay F., Lei H., Bajko J., Xu G., Li E., Chen T. (2009). KDM1B is a histone H3K4 demethylase required to establish maternal genomic imprints. Nature.

[bib20] Cloos P.A., Christensen J., Agger K., Maiolica A., Rappsilber J., Antal T., Hansen K.H., Helin K. (2006). The putative oncogene GASC1 demethylates tri- and dimethylated lysine 9 on histone H3. Nature.

[bib21] Coffey K., Rogerson L., Ryan-Munden C., Alkharaif D., Stockley J., Heer R., Sahadevan K., O'Neill D., Jones D., Darby S. (2013). The lysine demethylase, KDM4B, is a key molecule in androgen receptor signalling and turnover. Nucleic Acids Res..

[bib22] Cui J., Quan M., Xie D., Gao Y., Guha S., Fallon M.B., Chen J., Xie K. (2020). A novel KDM5A/MPC-1 signaling pathway promotes pancreatic cancer progression via redirecting mitochondrial pyruvate metabolism. Oncogene.

[bib23] D'Oto A., Fang J., Jin H., Xu B., Singh S., Mullasseril A., Jones V., Abu-Zaid A., von Buttlar X., Cooke B. (2021). KDM6B promotes activation of the oncogenic CDK4/6-pRB-E2F pathway by maintaining enhancer activity in MYCN-amplified neuroblastoma. Nat. Commun..

[bib24] Dambacher S., Hahn M., Schotta G. (2010). Epigenetic regulation of development by histone lysine methylation. Heredity.

[bib25] De Santa F., Totaro M.G., Prosperini E., Notarbartolo S., Testa G., Natoli G. (2007). The histone H3 lysine-27 demethylase Jmjd3 links inflammation to inhibition of polycomb-mediated gene silencing. Cell.

[bib26] Deng Y., Li M., Zhuo M., Guo P., Chen Q., Mo P., Li W., Yu C. (2021). Histone demethylase JMJD2D promotes the self-renewal of liver cancer stem-like cells by enhancing EpCAM and Sox9 expression. J. Biol. Chem..

[bib27] Dhar S.S., Alam H., Li N., Wagner K.W., Chung J., Ahn Y.W., Lee M.G. (2014). Transcriptional repression of histone deacetylase 3 by the histone demethylase KDM2A is coupled to tumorigenicity of lung cancer cells. J. Biol. Chem..

[bib28] Ding G., Xu X., Li D., Chen Y., Wang W., Ping D., Jia S., Cao L. (2020). Fisetin inhibits proliferation of pancreatic adenocarcinoma by inducing DNA damage via RFXAP/KDM4A-dependent histone H3K36 demethylation. Cell Death Dis..

[bib29] Duplaquet L., Li Y., Booker M.A., Xie Y., Olsen S.N., Patel R.A., Hong D., Hatton C., Denize T., Walton E. (2023). KDM6A epigenetically regulates subtype plasticity in small cell lung cancer. Nat. Cell Biol..

[bib30] Ezponda T., Dupere-Richer D., Will C.M., Small E.C., Varghese N., Patel T., Nabet B., Popovic R., Oyer J., Bulic M. (2017). UTX/KDM6A loss enhances the malignant phenotype of multiple myeloma and sensitizes cells to EZH2 inhibition. Cell Rep..

[bib31] Fan L., Peng G., Sahgal N., Fazli L., Gleave M., Zhang Y., Hussain A., Qi J. (2016). Regulation of c-Myc expression by the histone demethylase JMJD1A is essential for prostate cancer cell growth and survival. Oncogene.

[bib32] Fang R., Barbera A.J., Xu Y., Rutenberg M., Leonor T., Bi Q., Lan F., Mei P., Yuan G.C., Lian C. (2010). Human LSD2/KDM1b/AOF1 regulates gene transcription by modulating intragenic H3K4me2 methylation. Mol. Cell.

[bib33] Fang R., Chen F., Dong Z., Hu D., Barbera A.J., Clark E.A., Fang J., Yang Y., Mei P., Rutenberg M. (2013). LSD2/KDM1B and its cofactor NPAC/GLYR1 endow a structural and molecular model for regulation of H3K4 demethylation. Mol. Cell.

[bib34] Fang S., Zheng L., Shen L., Su Y., Ding J., Chen W., Chen X., Chen W., Shu G., Chen M. (2023). Inactivation of KDM5A suppresses growth and enhances chemosensitivity in liver cancer by modulating ROCK1/PTEN/AKT pathway. Eur. J. Pharmacol..

[bib35] Fang Z., Wang T.Q., Li H., Zhang G., Wu X.A., Yang L., Peng Y.L., Zou J., Li L.L., Xiang R. (2017). Discovery of pyrazolo[1,5-a]pyrimidine-3-carbonitrile derivatives as a new class of histone lysine demethylase 4D (KDM4D) inhibitors. Bioorg. Med. Chem. Lett..

[bib36] Feinberg A.P., Ohlsson R., Henikoff S. (2006). The epigenetic progenitor origin of human cancer. Nat. Rev. Genet..

[bib37] Forneris F., Binda C., Vanoni M.A., Mattevi A., Battaglioli E. (2005). Histone demethylation catalysed by LSD1 is a flavin-dependent oxidative process. FEBS Lett..

[bib38] Frescas D., Guardavaccaro D., Bassermann F., Koyama-Nasu R., Pagano M. (2007). JHDM1B/FBXL10 is a nucleolar protein that represses transcription of ribosomal RNA genes. Nature.

[bib39] Fu L.N., Wang Y.Q., Tan J., Xu J., Gao Q.Y., Chen Y.X., Fang J.Y. (2018). Role of JMJD2B in colon cancer cell survival under glucose-deprived conditions and the underlying mechanisms. Oncogene.

[bib40] Gao S., Chen S., Han D., Wang Z., Li M., Han W., Besschetnova A., Liu M., Zhou F., Barrett D. (2020). Chromatin binding of FOXA1 is promoted by LSD1-mediated demethylation in prostate cancer. Nat. Genet..

[bib41] Gerken P.A., Wolstenhulme J.R., Tumber A., Hatch S.B., Zhang Y., Muller S., Chandler S.A., Mair B., Li F., Nijman S.M.B. (2017). Discovery of a highly selective cell-active inhibitor of the histone lysine demethylases KDM2/7. Angew. Chem. Int. Ed. Engl..

[bib42] Gray Z.H., Chakraborty D., Duttweiler R.R., Alekbaeva G.D., Murphy S.E., Chetal K., Ji F., Ferman B.I., Honer M.A., Wang Z. (2023). Epigenetic balance ensures mechanistic control of MLL amplification and rearrangement. Cell.

[bib43] Guan T., Zhang Y., Li S., Zhang W., Song Y., Li Y., He Y., Chen Y. (2024). Discovery of an efficacious KDM5B PROTAC degrader GT-653 up-regulating IFN response genes in prostate cancer. Eur. J. Med. Chem..

[bib44] Guo X., Shi M., Sun L., Wang Y., Gui Y., Cai Z., Duan X. (2011). The expression of histone demethylase JMJD1A in renal cell carcinoma. Neoplasma.

[bib45] Harris W.J., Huang X., Lynch J.T., Spencer G.J., Hitchin J.R., Li Y., Ciceri F., Blaser J.G., Greystoke B.F., Jordan A.M. (2012). The histone demethylase KDM1A sustains the oncogenic potential of MLL-AF9 leukemia stem cells. Cancer Cell.

[bib46] Hashizume R., Andor N., Ihara Y., Lerner R., Gan H., Chen X., Fang D., Huang X., Tom M.W., Ngo V. (2014). Pharmacologic inhibition of histone demethylation as a therapy for pediatric brainstem glioma. Nat. Med..

[bib47] Hayami S., Yoshimatsu M., Veerakumarasivam A., Unoki M., Iwai Y., Tsunoda T., Field H.I., Kelly J.D., Neal D.E., Yamaue H. (2010). Overexpression of the JmjC histone demethylase KDM5B in human carcinogenesis: involvement in the proliferation of cancer cells through the E2F/RB pathway. Mol. Cancer.

[bib48] He J., Nguyen A.T., Zhang Y. (2011). KDM2b/JHDM1b, an H3K36me2-specific demethylase, is required for initiation and maintenance of acute myeloid leukemia. Blood.

[bib49] Hiatt J.B., Sandborg H., Garrison S.M., Arnold H.U., Liao S.Y., Norton J.P., Friesen T.J., Wu F., Sutherland K.D., Rienhoff H.Y. (2022). Inhibition of LSD1 with bomedemstat sensitizes small cell lung cancer to immune checkpoint blockade and T-cell killing. Clin. Cancer Res..

[bib50] Hinohara K., Wu H.J., Vigneau S., McDonald T.O., Igarashi K.J., Yamamoto K.N., Madsen T., Fassl A., Egri S.B., Papanastasiou M. (2018). KDM5 histone demethylase activity links cellular transcriptomic heterogeneity to therapeutic resistance. Cancer Cell.

[bib51] Hollebecque A., Salvagni S., Plummer R., Niccoli P., Capdevila J., Curigliano G., Moreno V., de Braud F., de Villambrosia S.G., Martin-Romano P. (2022). Clinical activity of CC-90011, an oral, potent, and reversible LSD1 inhibitor, in advanced malignancies. Cancer.

[bib52] Horton J.R., Liu X., Gale M., Wu L., Shanks J.R., Zhang X., Webber P.J., Bell J.S.K., Kales S.C., Mott B.T. (2016). Structural basis for KDM5A histone lysine demethylase inhibition by diverse compounds. Cell Chem. Biol..

[bib53] Horton J.R., Upadhyay A.K., Qi H.H., Zhang X., Shi Y., Cheng X. (2010). Enzymatic and structural insights for substrate specificity of a family of jumonji histone lysine demethylases. Nat. Struct. Mol. Biol..

[bib54] Hu F., Li H., Liu L., Xu F., Lai S., Luo X., Hu J., Yang X. (2018). Histone demethylase KDM4D promotes gastrointestinal stromal tumor progression through HIF1beta/VEGFA signalling. Mol. Cancer.

[bib55] Hu J., Sun T., Wang H., Chen Z., Wang S., Yuan L., Liu T., Li H.R., Wang P., Feng Y. (2016). MiR-215 is induced post-transcriptionally via HIF-Drosha complex and mediates glioma-initiating cell adaptation to hypoxia by targeting KDM1B. Cancer Cell.

[bib56] Huang J., Sengupta R., Espejo A.B., Lee M.G., Dorsey J.A., Richter M., Opravil S., Shiekhattar R., Bedford M.T., Jenuwein T. (2007). p53 is regulated by the lysine demethylase LSD1. Nature.

[bib57] Huang Y., Greene E., Murray Stewart T., Goodwin A.C., Baylin S.B., Woster P.M., Casero R.A. (2007). Inhibition of lysine-specific demethylase 1 by polyamine analogues results in reexpression of aberrantly silenced genes. Proc. Natl. Acad. Sci. U.S.A..

[bib58] Huang Z., Yang H. (2019). Upregulation of the long noncoding RNA ADPGK-AS1 promotes carcinogenesis and predicts poor prognosis in gastric cancer. Biochem. Biophys. Res. Commun..

[bib59] Hurst C.D., Alder O., Platt F.M., Droop A., Stead L.F., Burns J.E., Burghel G.J., Jain S., Klimczak L.J., Lindsay H. (2017). Genomic subtypes of non-invasive bladder cancer with distinct metabolic profile and female gender bias in KDM6A mutation frequency. Cancer Cell.

[bib60] Ismail T., Lee H.K., Kim C., Kwon T., Park T.J., Lee H.S. (2018). KDM1A microenvironment, its oncogenic potential, and therapeutic significance. Epigenetics Chromatin..

[bib61] Iwase S., Lan F., Bayliss P., de la Torre-Ubieta L., Huarte M., Qi H.H., Whetstine J.R., Bonni A., Roberts T.M., Shi Y. (2007). The X-linked mental retardation gene SMCX/JARID1C defines a family of histone H3 lysine 4 demethylases. Cell.

[bib62] Jia R., Yang L., Yuan X., Kong J., Liu Y., Yin W., Gao S., Zhang Y. (2019). GASC1 promotes stemness of esophageal squamous cell carcinoma via NOTCH1 promoter demethylation. J. Oncol..

[bib63] Johansson C., Velupillai S., Tumber A., Szykowska A., Hookway E.S., Nowak R.P., Strain-Damerell C., Gileadi C., Philpott M., Burgess-Brown N. (2016). Structural analysis of human KDM5B guides histone demethylase inhibitor development. Nat. Chem. Biol..

[bib64] Jones P.A., Baylin S.B. (2007). The epigenomics of cancer. Cell.

[bib65] Kahl P., Gullotti L., Heukamp L.C., Wolf S., Friedrichs N., Vorreuther R., Solleder G., Bastian P.J., Ellinger J., Metzger E. (2006). Androgen receptor coactivators lysine-specific histone demethylase 1 and four and a half LIM domain protein 2 predict risk of prostate cancer recurrence. Cancer Res..

[bib66] Kalisz M., Bernardo E., Beucher A., Maestro M.A., Del Pozo N., Millan I., Haeberle L., Schlensog M., Safi S.A., Knoefel W.T. (2020). HNF1A recruits KDM6A to activate differentiated acinar cell programs that suppress pancreatic cancer. EMBO J..

[bib67] Kaneko S., Li X. (2018). X chromosome protects against bladder cancer in females via a KDM6A-dependent epigenetic mechanism. Sci. Adv..

[bib68] Kang J.Y., Kim J.Y., Kim K.B., Park J.W., Cho H., Hahm J.Y., Chae Y.C., Kim D., Kook H., Rhee S. (2018). KDM2B is a histone H3K79 demethylase and induces transcriptional repression via sirtuin-1-mediated chromatin silencing. FASEB J..

[bib69] Kanouni T., Severin C., Cho R.W., Yuen N.Y., Xu J., Shi L., Lai C., Del Rosario J.R., Stansfield R.K., Lawton L.N. (2020). Discovery of CC-90011: a potent and selective reversible inhibitor of lysine specific demethylase 1 (LSD1). J. Med. Chem..

[bib70] Kim J.Y., Kim K.B., Eom G.H., Choe N., Kee H.J., Son H.J., Oh S.T., Kim D.W., Pak J.H., Baek H.J. (2012). KDM3B is the H3K9 demethylase involved in transcriptional activation of lmo2 in leukemia. Mol. Cell. Biol..

[bib71] Kim K., Lee J.M., Yu Y.S., Kim H., Nam H.J., Moon H.G., Noh D.Y., Kim K.I., Fang S., Baek S.H. (2017). RORalpha2 requires LSD1 to enhance tumor progression in breast cancer. Sci. Rep..

[bib72] Kim M.S., Cho H.I., Yoon H.J., Ahn Y.H., Park E.J., Jin Y.H., Jang Y.K. (2018). JIB-04, a small molecule histone demethylase inhibitor, selectively targets colorectal cancer stem cells by inhibiting the Wnt/beta-catenin signaling pathway. Sci. Rep..

[bib73] Klein B.J., Piao L., Xi Y., Rincon-Arano H., Rothbart S.B., Peng D., Wen H., Larson C., Zhang X., Zheng X. (2014). The histone-H3K4-specific demethylase KDM5B binds to its substrate and product through distinct PHD fingers. Cell Rep..

[bib74] Kontaki H., Talianidis I. (2010). Lysine methylation regulates E2F1-induced cell death. Mol. Cell.

[bib75] Kottakis F., Polytarchou C., Foltopoulou P., Sanidas I., Kampranis S.C., Tsichlis P.N. (2011). FGF-2 regulates cell proliferation, migration, and angiogenesis through an NDY1/KDM2B-miR-101-EZH2 pathway. Mol. Cell.

[bib76] Kruidenier L., Chung C.W., Cheng Z., Liddle J., Che K., Joberty G., Bantscheff M., Bountra C., Bridges A., Diallo H. (2012). A selective jumonji H3K27 demethylase inhibitor modulates the proinflammatory macrophage response. Nature.

[bib77] Kryukov G.V., Wilson F.H., Ruth J.R., Paulk J., Tsherniak A., Marlow S.E., Vazquez F., Weir B.A., Fitzgerald M.E., Tanaka M. (2016). MTAP deletion confers enhanced dependency on the PRMT5 arginine methyltransferase in cancer cells. Science.

[bib78] Kurt I.C., Sur I., Kaya E., Cingoz A., Kazancioglu S., Kahya Z., Toparlak O.D., Senbabaoglu F., Kaya Z., Ozyerli E. (2017). KDM2B, an H3K36-specific demethylase, regulates apoptotic response of GBM cells to TRAIL. Cell Death Dis..

[bib79] Kwon D.H., Kang J.Y., Joung H., Kim J.Y., Jeong A., Min H.K., Shin S., Lee Y.G., Kim Y.K., Seo S.B. (2021). SRF is a nonhistone methylation target of KDM2B and SET7 in the regulation of skeletal muscle differentiation. Exp. Mol. Med..

[bib80] Labbe R.M., Holowatyj A., Yang Z.Q. (2013). Histone lysine demethylase (KDM) subfamily 4: structures, functions and therapeutic potential. Am. J. Transl. Res..

[bib81] Laurent B., Ruitu L., Murn J., Hempel K., Ferrao R., Xiang Y., Liu S., Garcia B.A., Wu H., Wu F. (2015). A specific LSD1/KDM1A isoform regulates neuronal differentiation through H3K9 demethylation. Mol. Cell.

[bib82] Lee D.H., Kim G.W., Yoo J., Lee S.W., Jeon Y.H., Kim S.Y., Kang H.G., Kim D.H., Chun K.H., Choi J. (2021). Histone demethylase KDM4C controls tumorigenesis of glioblastoma by epigenetically regulating p53 and c-Myc. Cell Death Dis..

[bib83] Lee J.Y., Park J.H., Choi H.J., Won H.Y., Joo H.S., Shin D.H., Park M.K., Han B., Kim K.P., Lee T.J. (2017). LSD1 demethylates HIF1alpha to inhibit hydroxylation and ubiquitin-mediated degradation in tumor angiogenesis. Oncogene.

[bib84] Lee M.G., Norman J., Shilatifard A., Shiekhattar R. (2007). Physical and functional association of a trimethyl H3K4 demethylase and Ring6a/MBLR, a polycomb-like protein. Cell.

[bib85] Lee M.G., Villa R., Trojer P., Norman J., Yan K.P., Reinberg D., Di Croce L., Shiekhattar R. (2007). Demethylation of H3K27 regulates polycomb recruitment and H2A ubiquitination. Science.

[bib86] Lee M.G., Wynder C., Cooch N., Shiekhattar R. (2005). An essential role for CoREST in nucleosomal histone 3 lysine 4 demethylation. Nature.

[bib87] Lei Z.J., Wang J., Xiao H.L., Guo Y., Wang T., Li Q., Liu L., Luo X., Fan L.L., Lin L. (2015). Lysine-specific demethylase 1 promotes the stemness and chemoresistance of Lgr5(+) liver cancer initiating cells by suppressing negative regulators of beta-catenin signaling. Oncogene.

[bib88] Leng X., Wang J., An N., Wang X., Sun Y., Chen Z. (2020). Histone 3 lysine-27 demethylase KDM6A coordinates with KMT2B to play an oncogenic role in NSCLC by regulating H3K4me3. Oncogene.

[bib89] Li J., Lan Z., Liao W., Horner J.W., Xu X., Liu J., Yoshihama Y., Jiang S., Shim H.S., Slotnik M. (2023). Histone demethylase KDM5D upregulation drives sex differences in colon cancer. Nature.

[bib90] Li J., Yu B., Deng P., Cheng Y., Yu Y., Kevork K., Ramadoss S., Ding X., Li X., Wang C.Y. (2017). KDM3 epigenetically controls tumorigenic potentials of human colorectal cancer stem cells through Wnt/beta-catenin signalling. Nat. Commun..

[bib91] Li W., Zhao L., Zang W., Liu Z., Chen L., Liu T., Xu D., Jia J. (2011). Histone demethylase JMJD2B is required for tumor cell proliferation and survival and is overexpressed in gastric cancer. Biochem. Biophys. Res. Commun..

[bib92] Li Y., Zhang M., Sheng M., Zhang P., Chen Z., Xing W., Bai J., Cheng T., Yang F.C., Zhou Y. (2018). Therapeutic potential of GSK-J4, a histone demethylase KDM6B/JMJD3 inhibitor, for acute myeloid leukemia. J. Cancer Res. Clin. Oncol..

[bib93] Lim S., Janzer A., Becker A., Zimmer A., Schule R., Buettner R., Kirfel J. (2010). Lysine-specific demethylase 1 (LSD1) is highly expressed in ER-negative breast cancers and a biomarker predicting aggressive biology. Carcinogenesis.

[bib94] Lin G.H., Wu S.H., Ko Y.C., Lin C.H., Liao G.S., Chen T.W., Chen Y.J., Hsu K.F. (2023). Comprehensive analyses of prognostic values and immune infiltration of KDM3 gene family in hepatocellular carcinoma. Mol. Biotechnol..

[bib95] Lin T., Ponn A., Hu X., Law B.K., Lu J. (2010). Requirement of the histone demethylase LSD1 in Snai1-mediated transcriptional repression during epithelial-mesenchymal transition. Oncogene.

[bib96] Liu F., Wang Y., Yang Z., Cui X., Zheng L., Fu Y., Shao W., Zhang L., Yang Q., Jia J. (2022). KDM6B promotes gastric carcinogenesis and metastasis via upregulation of CXCR4 expression. Cell Death Dis..

[bib97] Liu W., Tanasa B., Tyurina O.V., Zhou T.Y., Gassmann R., Liu W.T., Ohgi K.A., Benner C., Garcia-Bassets I., Aggarwal A.K. (2010). PHF8 mediates histone H4 lysine 20 demethylation events involved in cell cycle progression. Nature.

[bib98] Loenarz C., Ge W., Coleman M.L., Rose N.R., Cooper C.D., Klose R.J., Ratcliffe P.J., Schofield C.J. (2010). PHF8, a gene associated with cleft lip/palate and mental retardation, encodes for an Nepsilon-dimethyl lysine demethylase. Hum. Mol. Genet..

[bib99] Lu B., Wei J., Zhou H., Chen J., Li Y., Ye L., Zhao W., Wu S. (2022). Histone H3K36me2 demethylase KDM2A promotes bladder cancer progression through epigenetically silencing RARRES3. Cell Death Dis..

[bib100] Luo H., Shenoy A.K., Li X., Jin Y., Jin L., Cai Q., Tang M., Liu Y., Chen H., Reisman D. (2016). MOF acetylates the histone demethylase LSD1 to suppress epithelial-to-mesenchymal transition. Cell Rep..

[bib101] Luo W., Chang R., Zhong J., Pandey A., Semenza G.L. (2012). Histone demethylase JMJD2C is a coactivator for hypoxia-inducible factor 1 that is required for breast cancer progression. Proc. Natl. Acad. Sci. U.S.A..

[bib102] Maes T., Mascaro C., Tirapu I., Estiarte A., Ciceri F., Lunardi S., Guibourt N., Perdones A., Lufino M.M.P., Somervaille T.C.P. (2018). ORY-1001, a potent and selective covalent KDM1A inhibitor, for the treatment of acute leukemia. Cancer Cell.

[bib103] Meng Z., Liu Y., Wang J., Fan H., Fang H., Li S., Yuan L., Liu C., Peng Y., Zhao W. (2020). Histone demethylase KDM7A is required for stem cell maintenance and apoptosis inhibition in breast cancer. J. Cell Physiol..

[bib104] Metzger E., Wissmann M., Yin N., Muller J.M., Schneider R., Peters A.H., Gunther T., Buettner R., Schule R. (2005). LSD1 demethylates repressive histone marks to promote androgen-receptor-dependent transcription. Nature.

[bib105] Mimasu S., Sengoku T., Fukuzawa S., Umehara T., Yokoyama S. (2008). Crystal structure of histone demethylase LSD1 and tranylcypromine at 2.25 A. Biochem. Biophys. Res. Commun..

[bib106] Mohammad H.P., Smitheman K.N., Kamat C.D., Soong D., Federowicz K.E., Van Aller G.S., Schneck J.L., Carson J.D., Liu Y., Butticello M. (2015). A DNA hypomethylation signature predicts antitumor activity of LSD1 inhibitors in SCLC. Cancer Cell.

[bib107] Mokou M., Klein J., Makridakis M., Bitsika V., Bascands J.L., Saulnier-Blache J.S., Mullen W., Sacherer M., Zoidakis J., Pieske B. (2019). Proteomics based identification of KDM5 histone demethylases associated with cardiovascular disease. EBioMedicine.

[bib108] Muntean A.G., Hess J.L. (2009). Epigenetic dysregulation in cancer. Am. J. Pathol..

[bib109] Ng S.S., Kavanagh K.L., McDonough M.A., Butler D., Pilka E.S., Lienard B.M., Bray J.E., Savitsky P., Gileadi O., von Delft F. (2007). Crystal structures of histone demethylase JMJD2A reveal basis for substrate specificity. Nature.

[bib110] Osawa T., Muramatsu M., Wang F., Tsuchida R., Kodama T., Minami T., Shibuya M. (2011). Increased expression of histone demethylase JHDM1D under nutrient starvation suppresses tumor growth via down-regulating angiogenesis. Proc. Natl. Acad. Sci. U.S.A..

[bib111] Park D.H., Hong S.J., Salinas R.D., Liu S.J., Sun S.W., Sgualdino J., Testa G., Matzuk M.M., Iwamori N., Lim D.A. (2014). Activation of neuronal gene expression by the JMJD3 demethylase is required for postnatal and adult brain neurogenesis. Cell Rep..

[bib112] Peng K., Kou L., Yu L., Bai C., Li M., Mo P., Li W., Yu C. (2019). Histone demethylase JMJD2D interacts with beta-catenin to induce transcription and activate colorectal cancer cell proliferation and tumor growth in mice. Gastroenterology.

[bib113] Peng K., Zhuo M., Li M., Chen Q., Mo P., Yu C. (2020). Histone demethylase JMJD2D activates HIF1 signaling pathway via multiple mechanisms to promote colorectal cancer glycolysis and progression. Oncogene.

[bib114] Qi H.H., Sarkissian M., Hu G.Q., Wang Z., Bhattacharjee A., Gordon D.B., Gonzales M., Lan F., Ongusaha P.P., Huarte M. (2010). Histone H4K20/H3K9 demethylase PHF8 regulates zebrafish brain and craniofacial development. Nature.

[bib115] Ramadoss S., Chen X., Wang C.Y. (2012). Histone demethylase KDM6B promotes epithelial-mesenchymal transition. J. Biol. Chem..

[bib116] Ramakrishnan S., Granger V., Rak M., Hu Q., Attwood K., Aquila L., Krishnan N., Osiecki R., Azabdaftari G., Guru K. (2019). Inhibition of EZH2 induces NK cell-mediated differentiation and death in muscle-invasive bladder cancer. Cell Death Differ..

[bib117] Ramanan R., Chaturvedi S.S., Lehnert N., Schofield C.J., Karabencheva-Christova T.G., Christov C.Z. (2020). Catalysis by the JmjC histone demethylase KDM4A integrates substrate dynamics, correlated motions and molecular orbital control. Chem. Sci..

[bib118] Rissi V.B., Glanzner W.G., De Macedo M.P., Gutierrez K., Baldassarre H., Goncalves P.B.D., Bordignon V. (2019). The histone lysine demethylase KDM7A is required for normal development and first cell lineage specification in porcine embryos. Epigenetics.

[bib119] Roy N., Hebrok M. (2015). Regulation of cellular identity in cancer. Dev. Cell.

[bib120] Schiller R., Scozzafava G., Tumber A., Wickens J.R., Bush J.T., Rai G., Lejeune C., Choi H., Yeh T.L., Chan M.C. (2014). A cell-permeable ester derivative of the JmjC histone demethylase inhibitor IOX1. ChemMedChem.

[bib121] Schotta G., Lachner M., Sarma K., Ebert A., Sengupta R., Reuter G., Reinberg D., Jenuwein T. (2004). A silencing pathway to induce H3-K9 and H4-K20 trimethylation at constitutive heterochromatin. Genes Dev..

[bib122] Schulte J.H., Lim S., Schramm A., Friedrichs N., Koster J., Versteeg R., Ora I., Pajtler K., Klein-Hitpass L., Kuhfittig-Kulle S. (2009). Lysine-specific demethylase 1 is strongly expressed in poorly differentiated neuroblastoma: implications for therapy. Cancer Res..

[bib123] Seligson D.B., Horvath S., Shi T., Yu H., Tze S., Grunstein M., Kurdistani S.K. (2005). Global histone modification patterns predict risk of prostate cancer recurrence. Nature.

[bib124] Shao G., Wang J., Li Y., Liu X., Xie X., Wan X., Yan M., Jin J., Lin Q., Zhu H. (2015). Lysine-specific demethylase 1 mediates epidermal growth factor signaling to promote cell migration in ovarian cancer cells. Sci. Rep..

[bib125] Shen H.F., Zhang W.J., Huang Y., He Y.H., Hu G.S., Wang L., Peng B.L., Yi J., Li T.T., Rong R. (2021). The dual function of KDM5C in both gene transcriptional activation and repression promotes breast cancer cell growth and tumorigenesis. Adv. Sci..

[bib126] Shi L., Sun L., Li Q., Liang J., Yu W., Yi X., Yang X., Li Y., Han X., Zhang Y. (2011). Histone demethylase JMJD2B coordinates H3K4/H3K9 methylation and promotes hormonally responsive breast carcinogenesis. Proc. Natl. Acad. Sci. U.S.A..

[bib127] Shi Y., Lan F., Matson C., Mulligan P., Whetstine J.R., Cole P.A., Casero R.A., Shi Y. (2004). Histone demethylation mediated by the nuclear amine oxidase homolog LSD1. Cell.

[bib128] Shin S., Janknecht R. (2007). Activation of androgen receptor by histone demethylases JMJD2A and JMJD2D. Biochem. Biophys. Res. Commun..

[bib129] Soldi R., Ghosh Halder T., Weston A., Thode T., Drenner K., Lewis R., Kaadige M.R., Srivastava S., Daniel Ampanattu S., Rodriguez Del Villar R. (2020). The novel reversible LSD1 inhibitor SP-2577 promotes anti-tumor immunity in SWItch/Sucrose-NonFermentable (SWI/SNF) complex mutated ovarian cancer. PLoS One.

[bib130] Song T., Lv S., Ma X., Zhao X., Fan L., Zou Q., Li N., Yan Y., Zhang W., Sun L. (2023). TRIM28 represses renal cell carcinoma cell proliferation by inhibiting TFE3/KDM6A-regulated autophagy. J. Biol. Chem..

[bib131] Spangle J.M., Dreijerink K.M., Groner A.C., Cheng H., Ohlson C.E., Reyes J., Lin C.Y., Bradner J., Zhao J.J., Roberts T.M. (2016). PI3K/AKT signaling regulates H3K4 methylation in breast cancer. Cell Rep..

[bib132] Stender J.D., Pascual G., Liu W., Kaikkonen M.U., Do K., Spann N.J., Boutros M., Perrimon N., Rosenfeld M.G., Glass C.K. (2012). Control of proinflammatory gene programs by regulated trimethylation and demethylation of histone H4K20. Mol. Cell.

[bib133] Sun Q., Zhuang Z., Bai R., Deng J., Xin T., Zhang Y., Li Q., Han B. (2023). Lysine 68 methylation-dependent SOX9 stability control modulates chondrogenic differentiation in dental pulp stem cells. Adv. Sci..

[bib134] Tang D., He J., Dai Y., Geng X., Leng Q., Jiang H., Sun R., Xu S. (2022). Targeting KDM1B-dependent miR-215-AR-AGR2-axis promotes sensitivity to enzalutamide-resistant prostate cancer. Cancer Gene Ther..

[bib135] Terashima M., Ishimura A., Wanna-Udom S., Suzuki T. (2017). Epigenetic regulation of epithelial-mesenchymal transition by KDM6A histone demethylase in lung cancer cells. Biochem. Biophys. Res. Commun..

[bib136] Torres I.O., Kuchenbecker K.M., Nnadi C.I., Fletterick R.J., Kelly M.J., Fujimori D.G. (2015). Histone demethylase KDM5A is regulated by its reader domain through a positive-feedback mechanism. Nat. Commun..

[bib137] Tseng L.L., Cheng H.H., Yeh T.S., Huang S.C., Syu Y.Y., Chuu C.P., Yuh C.H., Kung H.J., Wang W.C. (2020). Targeting the histone demethylase PHF8-mediated PKCalpha-Src-PTEN axis in HER2-negative gastric cancer. Proc. Natl. Acad. Sci. U.S.A..

[bib138] Tsukada Y., Fang J., Erdjument-Bromage H., Warren M.E., Borchers C.H., Tempst P., Zhang Y. (2006). Histone demethylation by a family of JmjC domain-containing proteins. Nature.

[bib139] Tu S., Bulloch E.M., Yang L., Ren C., Huang W.C., Hsu P.H., Chen C.H., Liao C.L., Yu H.M., Lo W.S. (2007). Identification of histone demethylases in *Saccharomyces cerevisiae*. J. Biol. Chem..

[bib140] Tumber A., Nuzzi A., Hookway E.S., Hatch S.B., Velupillai S., Johansson C., Kawamura A., Savitsky P., Yapp C., Szykowska A. (2017). Potent and selective KDM5 inhibitor stops cellular demethylation of H3K4me3 at transcription start sites and proliferation of MM1S myeloma cells. Cell Chem. Biol..

[bib141] Tzatsos A., Pfau R., Kampranis S.C., Tsichlis P.N. (2009). Ndy1/KDM2B immortalizes mouse embryonic fibroblasts by repressing the Ink4a/Arf locus. Proc. Natl. Acad. Sci. U.S.A..

[bib142] Uemura M., Yamamoto H., Takemasa I., Mimori K., Hemmi H., Mizushima T., Ikeda M., Sekimoto M., Matsuura N., Doki Y. (2010). Jumonji domain containing 1A is a novel prognostic marker for colorectal cancer: in vivo identification from hypoxic tumor cells. Clin. Cancer Res..

[bib143] Vinogradova M., Gehling V.S., Gustafson A., Arora S., Tindell C.A., Wilson C., Williamson K.E., Guler G.D., Gangurde P., Manieri W. (2016). An inhibitor of KDM5 demethylases reduces survival of drug-tolerant cancer cells. Nat. Chem. Biol..

[bib144] Wade M.A., Jones D., Wilson L., Stockley J., Coffey K., Robson C.N., Gaughan L. (2015). The histone demethylase enzyme KDM3A is a key estrogen receptor regulator in breast cancer. Nucleic Acids Res..

[bib145] Walport L.J., Hopkinson R.J., Vollmar M., Madden S.K., Gileadi C., Oppermann U., Schofield C.J., Johansson C. (2014). Human UTY(KDM6C) is a male-specific Nϵ-methyl lysyl demethylase. J. Biol. Chem..

[bib146] Wang D., Han S., Peng R., Jiao C., Wang X., Yang X., Yang R., Li X. (2016). Depletion of histone demethylase KDM5B inhibits cell proliferation of hepatocellular carcinoma by regulation of cell cycle checkpoint proteins p15 and p27. J. Exp. Clin. Cancer Res..

[bib147] Wang D., Zhang Y., Liao Z., Ge H., Gungor C., Li Y. (2023). KDM5 family of demethylases promotes CD44-mediated chemoresistance in pancreatic adenocarcinomas. Sci. Rep..

[bib148] Wang H., Song C., Ding Y., Pan X., Ge Z., Tan B.H., Gowda C., Sachdev M., Muthusami S., Ouyang H. (2016). Transcriptional regulation of JARID1B/KDM5B histone demethylase by ikaros, histone deacetylase 1 (HDAC1), and casein kinase 2 (CK2) in B-cell acute lymphoblastic leukemia. J. Biol. Chem..

[bib149] Wang J., Telese F., Tan Y., Li W., Jin C., He X., Basnet H., Ma Q., Merkurjev D., Zhu X. (2015). LSD1n is an H4K20 demethylase regulating memory formation via transcriptional elongation control. Nat. Neurosci..

[bib150] Wang J., Zhang Z.Y., Jiang J., Tang L., Wang X.Y., Wang Z., Yang X.L., Yu X.L., Huang C.C., Chen F. (2022). KDM2A plays a dual role in regulating the expression of malignancy-related genes in esophageal squamous cell carcinoma. Biochem. Biophys. Res. Commun..

[bib151] Wang Y., Liu S., Wang Y., Li B., Liang J., Chen Y., Tang B., Yu S., Wang H. (2024). KDM5B promotes SMAD4 loss-driven drug resistance through activating DLG1/YAP to induce lipid accumulation in pancreatic ductal adenocarcinoma. Cell Death Discov..

[bib152] Wang Y., Zhang H., Chen Y., Sun Y., Yang F., Yu W., Liang J., Sun L., Yang X., Shi L. (2009). LSD1 is a subunit of the NuRD complex and targets the metastasis programs in breast cancer. Cell.

[bib153] Wanna-Udom S., Terashima M., Suphakhong K., Ishimura A., Takino T., Suzuki T. (2021). KDM2B is involved in the epigenetic regulation of TGF-beta-induced epithelial-mesenchymal transition in lung and pancreatic cancer cell lines. J. Biol. Chem..

[bib154] Whetstine J.R., Nottke A., Lan F., Huarte M., Smolikov S., Chen Z., Spooner E., Li E., Zhang G., Colaiacovo M. (2006). Reversal of histone lysine trimethylation by the JMJD2 family of histone demethylases. Cell.

[bib155] Wissmann M., Yin N., Muller J.M., Greschik H., Fodor B.D., Jenuwein T., Vogler C., Schneider R., Gunther T., Buettner R. (2007). Cooperative demethylation by JMJD2C and LSD1 promotes androgen receptor-dependent gene expression. Nat. Cell Biol..

[bib156] Xiang Y., Zhu Z., Han G., Ye X., Xu B., Peng Z., Ma Y., Yu Y., Lin H., Chen A.P. (2007). JARID1B is a histone H3 lysine 4 demethylase up-regulated in prostate cancer. Proc. Natl. Acad. Sci. U.S.A..

[bib157] Xiao Z., Yang X., Liu Z., Shao Z., Song C., Zhang K., Wang X., Li Z. (2021). GASC1 promotes glioma progression by enhancing NOTCH1 signaling. Mol. Med. Rep..

[bib158] Xu X., Wang L., Hu L., Dirks W.G., Zhao Y., Wei Z., Chen D., Li Z., Wang Z., Han Y. (2020). Small molecular modulators of JMJD1C preferentially inhibit growth of leukemia cells. Int. J. Cancer.

[bib159] Xu Z., Xia Y., Xiao Z., Jia Y., Li L., Jin Y., Zhao Q., Wan L., Yi T., Yu Y. (2019). Comprehensive profiling of JMJD3 in gastric cancer and its influence on patient survival. Sci. Rep..

[bib160] Yamada D., Kobayashi S., Yamamoto H., Tomimaru Y., Noda T., Uemura M., Wada H., Marubashi S., Eguchi H., Tanemura M. (2012). Role of the hypoxia-related gene, JMJD1A, in hepatocellular carcinoma: clinical impact on recurrence after hepatic resection. Ann. Surg. Oncol..

[bib161] Yamamoto S., Wu Z., Russnes H.G., Takagi S., Peluffo G., Vaske C., Zhao X., Moen Vollan H.K., Maruyama R., Ekram M.B. (2014). JARID1B is a luminal lineage-driving oncogene in breast cancer. Cancer Cell.

[bib162] Yamane K., Tateishi K., Klose R.J., Fang J., Fabrizio L.A., Erdjument-Bromage H., Taylor-Papadimitriou J., Tempst P., Zhang Y. (2007). PLU-1 is an H3K4 demethylase involved in transcriptional repression and breast cancer cell proliferation. Mol. Cell.

[bib163] Yan H., Zhu L., Zhang J., Lin Z. (2021). Histone demethylase KDM4D inhibition suppresses renal cancer progression and angiogenesis through JAG1 signaling. Cell Death Discov..

[bib164] Yang G.J., Ko C.N., Zhong H.J., Leung C.H., Ma D.L. (2019). Structure-based discovery of a selective KDM5A inhibitor that exhibits anti-cancer activity via inducing cell cycle arrest and senescence in breast cancer cell lines. Cancers.

[bib165] Yang H., Kwon C.S., Choi Y., Lee D. (2016). Both H4K20 mono-methylation and H3K56 acetylation mark transcription-dependent histone turnover in fission yeast. Biochem. Biophys. Res. Commun..

[bib166] Yang J., Huang J., Dasgupta M., Sears N., Miyagi M., Wang B., Chance M.R., Chen X., Du Y., Wang Y. (2010). Reversible methylation of promoter-bound STAT3 by histone-modifying enzymes. Proc. Natl. Acad. Sci. U.S.A..

[bib167] Yoshihama Y., LaBella K.A., Kim E., Bertolet L., Colic M., Li J., Shang X., Wu C.J., Spring D.J., Wang Y.A. (2021). AR-negative prostate cancer is vulnerable to loss of JMJD1C demethylase. Proc. Natl. Acad. Sci. U.S.A..

[bib168] Zaman S.U., Pagare P.P., Ma H., Hoyle R.G., Zhang Y., Li J. (2024). Novel PROTAC probes targeting KDM3 degradation to eliminate colorectal cancer stem cells through inhibition of Wnt/beta-catenin signaling. RSC Med. Chem..

[bib169] Zeng Z., Li Z., Xue J., Xue H., Liu Z., Zhang W., Liu H., Xu S. (2023). KDM4C silencing inhibits cell migration and enhances radiosensitivity by inducing CXCL2 transcription in hepatocellular carcinoma. Cell Death Discov..

[bib170] Zhang W., Cheng J., Diao P., Wang D., Zhang W., Jiang H., Wang Y. (2020). Therapeutically targeting head and neck squamous cell carcinoma through synergistic inhibition of LSD1 and JMJD3 by TCP and GSK-J1. Br. J. Cancer.

[bib171] Zhang W., Sviripa V.M., Xie Y., Yu T., Haney M.G., Blackburn J.S., Adeniran C.A., Zhan C.G., Watt D.S., Liu C. (2020). Epigenetic regulation of Wnt signaling by carboxamide-substituted benzhydryl amines that function as histone demethylase inhibitors. iScience.

[bib172] Zhang Z., Chen B., Zhu Y., Zhang T., Zhang X., Yuan Y., Xu Y. (2021). The Jumonji domain-containing histone demethylase homolog 1D/lysine demethylase 7A (JHDM1D/KDM7A) is an epigenetic activator of RHOJ transcription in breast cancer cells. Front. Cell Dev. Biol..

[bib173] Zhao B., Liang Q., Ren H., Zhang X., Wu Y., Zhang K., Ma L.Y., Zheng Y.C., Liu H.M. (2020). Discovery of pyrazole derivatives as cellular active inhibitors of histone lysine specific demethylase 5B (KDM5B/JARID1B). Eur. J. Med. Chem..

[bib174] Zhou Q., Zhang Y., Wang B., Zhou W., Bi Y., Huai W., Chen X., Chen Y., Liu Z., Liu X. (2020). KDM2B promotes IL-6 production and inflammatory responses through Brg1-mediated chromatin remodeling. Cell. Mol. Immunol..

[bib175] Zhu W., Ding Y., Huang W., Guo N., Ren Q., Wang N., Ma X. (2024). Synergistic effects of the KDM4C inhibitor SD70 and the menin inhibitor MI-503 against MLL::AF9-driven acute myeloid leukaemia. Br. J. Haematol..

